# A long-living species of the hydrophiloid beetles: Helophorus sibiricus from the early Miocene deposits of Kartashevo (Siberia, Russia)

**DOI:** 10.3897/zookeys.130.1378

**Published:** 2011-09-24

**Authors:** Martin Fikáček, Alexander Prokin, Robert B. Angus

**Affiliations:** 1 Department of Entomology, National Museum, Kunratice 1, CZ-148 00 Praha 2, Czech Republic; 2 Department of Zoology, Faculty of Science, Charles University in Prague, Viničná 7, CZ-128 44 Praha 2, Czech Republic; 3 Voronezh State University, Research-Educational Center “Venevitinovo”, Universitetskaya sq. 1, Voronezh 394006, Russia; 4 Department of Entomology, The Natural History Museum, Cromwell Road, London SW7 5BD, UK

**Keywords:** Coleoptera, Hydrophiloidea, *Helophorus*, Miocene, Kartashevo, long-living species, environmental stability

## Abstract

The recent hydrophiloid species *Helophorus (Gephelophorus) sibiricus* (Motschulsky, 1860) is recorded from the early Miocene deposits of Kartashevo assigned to the Ombinsk Formation. A detailed comparison with recent specimens allowed a confident identification of the fossil specimen, which is therefore the oldest record of a recent species for the Hydrophiloidea. The paleodistribution as well as recent distribution of the species is summarized, and the relevance of the fossil is discussed. In addition, the complex geological settings of the Kartashevo area are briefly summarized.

## Introduction

The understanding of the evolution of beetles in the late Cenozoic has changed drastically within recent decades. This concerns especially the Pleistocene beetles preserved as unmineralized subfossil specimens. Originally, the remains from the Pleistocene peatbog or asphalt deposits were assigned to extinct species by historical authors (e.g. Łomnicki 1894, Grinell 1908, Orchymont 1927, Pierce 1946, 1948, 1949, 1954, Scudder 1900), supporting the idea of a high evolutionary rate induced by the climate changes during the Pleistocene. Later, more detailed studies of subfossil specimens sometimes based even on the study of their well-preserved genitalia revealed that the majority of Pleistocene subfossil beetles belong to recent species (Elias 2010; for examples of taxonomic revisions see e.g., Darlington 1938, Angus 1973, 1997, Miller and Peck 1979, Doyen and Miller 1980, Miller et al. 1981) and resulted in the Pleistocene evolutionary stasis paradigm (Coope 1970, 2004). Recent studies of many beetle groups based on molecular data contradict the results based on the fossil record for many beetle groups and indicate a high evolutionary rate during the Pleistocene (e.g., Ribera and Vogler 2004, Cardoso and Vogler 2005, Goméz-Zurita et al. 2007, Borer et al. 2010, Ribera and Faille 2010). This disagreement of molecular and fossil results is interpreted as resulting from fossil data being only available for high latitude areas (Ribera and Vogler 2004, Abellán et al. 2011).

The presence of recent species in Pleistocene deposits invites the question as to whether the same might not be the case even in older, Pliocene or Miocene deposits. Pre-Pleistocene beetle fossils were mostly assigned to extinct species of recent genera (Scudder 1891, 1900, Handlirsch 1908). However, this traditional view has been contradicted recently by many molecular studies showing the pre-Pleistocene origin of various beetle species (e.g., Gómez-Zurita et al 2007, Sota et al. 2008, Papadopoulou et al. 2009, Ribera et al. 2010a, b, Stüben and Astrin 2010). The fossil record reliably supporting the hypothesis of long-living species is, however, rather scarce so far (Matthews 1977, Grebennikov 2010, Hörnschemeyer et al. 2010) as most published data are based on approximate identification of fragmentary remains only (e.g., Matthews 1977, Bennike and Böcher 1990, Matthews et al. 2003).

Representatives of the hydrophiloid genus *Helophorus* Fabricius, 1775 are frequently used in the studies of Quaternary beetle communities. The taxonomy as well as recent distribution of most species is well known due to the studies by the third author (largely summarized by Angus (1997)) and Smetana (1985), and reliable species identification is often possible even without the examination of male genitalia, using the external characters as e.g. sculpture and shape of the pronotum, width of pronotal flanks and morphology of the elytron. These characters often allow recognition of very similar sibling species which makes *Helophorus* one of the best model beetle taxa for evaluating the changes of beetle faunas during the Pleistocene (e.g. Angus 1973, 1997, Morgan 1989, Elias 2010). Ca. 45 species of *Helophorus* were recorded from the latest Pliocene, Pleistocene and Holocene subfossil deposits so far (Scudder 1890, Morgan and Morgan 1980, Buckland and Buckland 2006), all of them belonging to recent species (the only exception is *Helophorus rigescens* Scudder, 1890 whose revision is impossible as its type specimen is lost; Fikáček, unpubl. data). Four extinct species were described from the late Miocene deposits in Alaska (2 spp.) and southern Germany (2 spp.) by Matthews (1976) and Heer (1862). No other fossils of the genus are known from older Tertiary deposits.

Detailed examination of a well-preserved Miocene *Helophorus* fossil from the collection of the Paleontological Institute in Moscow revealed that it may be reliably assigned to the living species *Helophorus sibiricus* and represents therefore the oldest record of recent species for hydrophiloid beetles. The results of the studies of this fossil are summarized within this paper and the relevance of the record is discussed.

We would like to dedicate this contribution to Alexandr P. Rasnitsyn on the occasion of his 75th birthday as our thanks for his outstanding contribution to the paleontology and entomology and his massive support of younger generations of entomologists and paleontologists all over the world. We wish him many more scientifically productive years full of good health and cheerfulness!

## Geological setting

The geology of the area around Kartashevo village on the right bank of the Irtysh river (56°06'54"N, 74°41'27"E) is rather complex especially in the eroded parts where two formations of different age are in contact: the older Abrosimovka Formation and the overlying Beshcheul Formation.

The Abrosimovka Formation was dated recently by the comparison of its palynological spectra with the Upper Baygubek Subhorizon of Aral and the North Ustyurt by Zykin (2009), and its age was considered as upper Oligocene. This was in agreement with the opinion of Dorofeev (1963) who dated the fossil flora of the Abrosimovka Formation to the upper Oligocene as it retains the basic structure of the Lagernosad-Rezhenka floras with many archaic elements. Alternative dating was proposed by Volkova et al. (2001) who dated the Abrosimovka Horizon to the lower Miocene (Aquitanian–Burdigalian).

The overlying Beshcheul Formation was dated as middle Miocene by Dorofeev (1963) due to the similarities of its fossil flora with the Sarmatian floras of the Russian plain. Middle Miocene was adopted as the age of the formation even in more recent publications (e.g., Volkova 1982, LePage 2007, Durnikin 2010).

When describing the fossil flora of the Kartashevo region, Dorofeev (1963) recognized five layers in the coastal section of the Irtysh river (listed from deeper parts towards the current surface): (1) the outputs of lignite, (2) the horizon of the blue-gray, very dense clay with layers of plant detritus 0–5 m from the water edge (the Kartashevo clay stratum), (3) the characteristically stratified suglino-loam of the Beshcheul Formation with layers of plant detritus ca, between 14–16 m from the water edge, (4) sand without plant residues, possibly related to the Ishim Formation, and (5) soil and a thick layer of compost. The fossil specimen refered in this paper was collected from the exposed Kartashevo clay stratum (i.e., layer 2 sensu Dorofeev (1963)) on the right bank of the Irtysh River under and just above the water edge (E. K. Sychevskaya, pers. comm.). The clay stratum was originally assigned to the Abrosimovka Formation and therefore refered as upper Oligocene in age e.g. by Sukacheva (1982). Recently, it was found to represent a separate Ombinsk Formation overlying the Abrosimovka Formation and underlying the Beshcheul Formation in the Kartashevo area. The Ombinsk Formation is currently dated to the lower Miocene (V.S. Zykin, pers. comm.). The samples of spores, pollen and dinocysts from the stratum were analyzed by M. A. Akhmet’ev and N. I. Zaporozhets and confirmed the lower Miocene origin of the stratum (E. K. Sychevskaya, pers. comm.). The Oligocene-Miocene boundary is currently placed between the Abrosimovka and Ombinsk Formations in western Siberia (Zykin 2009), which also corresponds well with the lower Miocene age of the Kartashevo clay stratum. Hence, the fossil presented in this paper may be realiably assigned to the Burdigalian or Aquitalian stages, and approximately dated as 16–23 million years old. Zykin (2009) mentioned that the climate was relatively stable, moderately warm and rather humid in the area on the Oligocene-Miocene boundary.

Only one insect species was previously known from the Kartashevo clay stratum – the caddisfly case described as *Terrindusia* (s.str.) *eugeniae* Sukatcheva, 1982 (originally assigned to the Abrosimovka Formation by Sukatcheva (1982) as the Ombinsk Formation was not recognized at that time). Besides the *Helophorus* fossil described in detail within this paper, there is also another fragmentary fossil from this stratum (PIN 3285/6) which may belong to the hydrophilid genera *Hydrochara* Berthold, 1827, *Hydrobiomorpha* Blackburn, 1888or *Brownephilus* Mouchamps, 1959based on preserved morphological characters. More detailed identification is not possible and the fossil is therefore not treated further in this paper.

## Material and methods

The fossil specimen presented in this paper was examined in dry condition. Habitus photographs of both fossil and recent specimens were taken using the Canon D-550 digital camera with attached Canon MP-E65mm f/2.8 1–5X macrolens, drawings were traced from the photographs along with a simultaneous check of the fossil specimen. Scaning electron micrographs of fossil as well as recent specimens were prepared using the Hitachi S-3700N environmental electron microscope in the Department of Entomology, National Museum in Prague. Data on the morphology of recent *Helophorus sibiricus* are based on the specimens deposited at the Department of Entomology, National Museum in Prague, and the Natural History Museum in London.

The Pleistocene records of *Helophorus sibiricus* from Europe were taken from the BugsCEP database available on-line (Buckland and Buckland 2006; data file updated on 18th January 2011), data on records in Siberia and North America were taken from the published papers (Morgan and Morgan 1980, Garry et al. 1990, Andreev et al. 2004, Sher et al. 2005, Elias et al. 2006). Published Holocene subfossil records are not considered in this paper and are also omitted in Fig. 10 as they are too recent and therefore not relevant to the topic of this paper; moreover, they mostly fall into the recent distribution range of the species. Data on the recent distribution were adopted from the papers by (Angus (1973, 1992), Smetana (1985) and Hansen (2004) and the species range was slightly adapted according to the unpublished faunistic data known to the authors.

## Results

### Superfamily Hydrophiloidea. Family Helophoridae

#### Helophorus (Gephelophorus) sibiricus

(Motschulsky, 1860)

http://species-id.net/wiki/Helophorus_(Gephelophorus)_sibiricus

Empleurus sibiricus Motschulsky, 1860: 104 – Type locality: recent: Russia, East Siberia, “Tourkinsk” [=Turka] at Lake Baikal.
Helophorus sibiricus (Motschulsky): transferred to *Helophorus* by Gemminger and Harold (1868).
For complete synonymy see Hansen (1999).

##### Nomenclature citation.

For complete synonymy see Hansen (1999).

##### Material examined.

PIN 3285/5 (piece and counterpiece): Russia, Omsk region, Western Siberia, right bank of Irtysh river at Kartashevo village [56°6'54.11"N, 74°41'27.20"E], leg. E. K. Sychevskaya 1966. Ombinsk Formation, early Miocene, ca. 23–16 million years ago. Deposited in the collection of the A.A. Borissiak Paleontological Institute of the Russian Academy of Sciences, Moscow, Russia.

##### Description of the fossil

**Figures 1–2.**
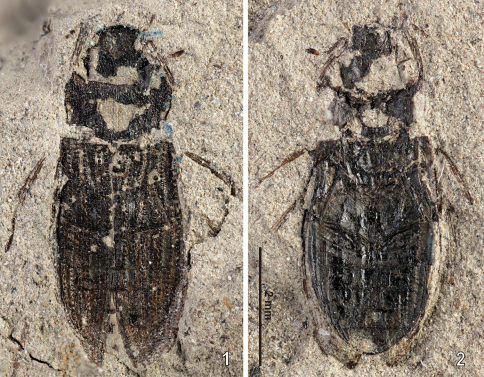
*Helophorus sibiricus* (Motschulsky, 1860), photo of the early Miocene fossil No. PIN 3285/5 from Kartashevo **1** piece **2** counterpiece.

**Figures 3–4.**
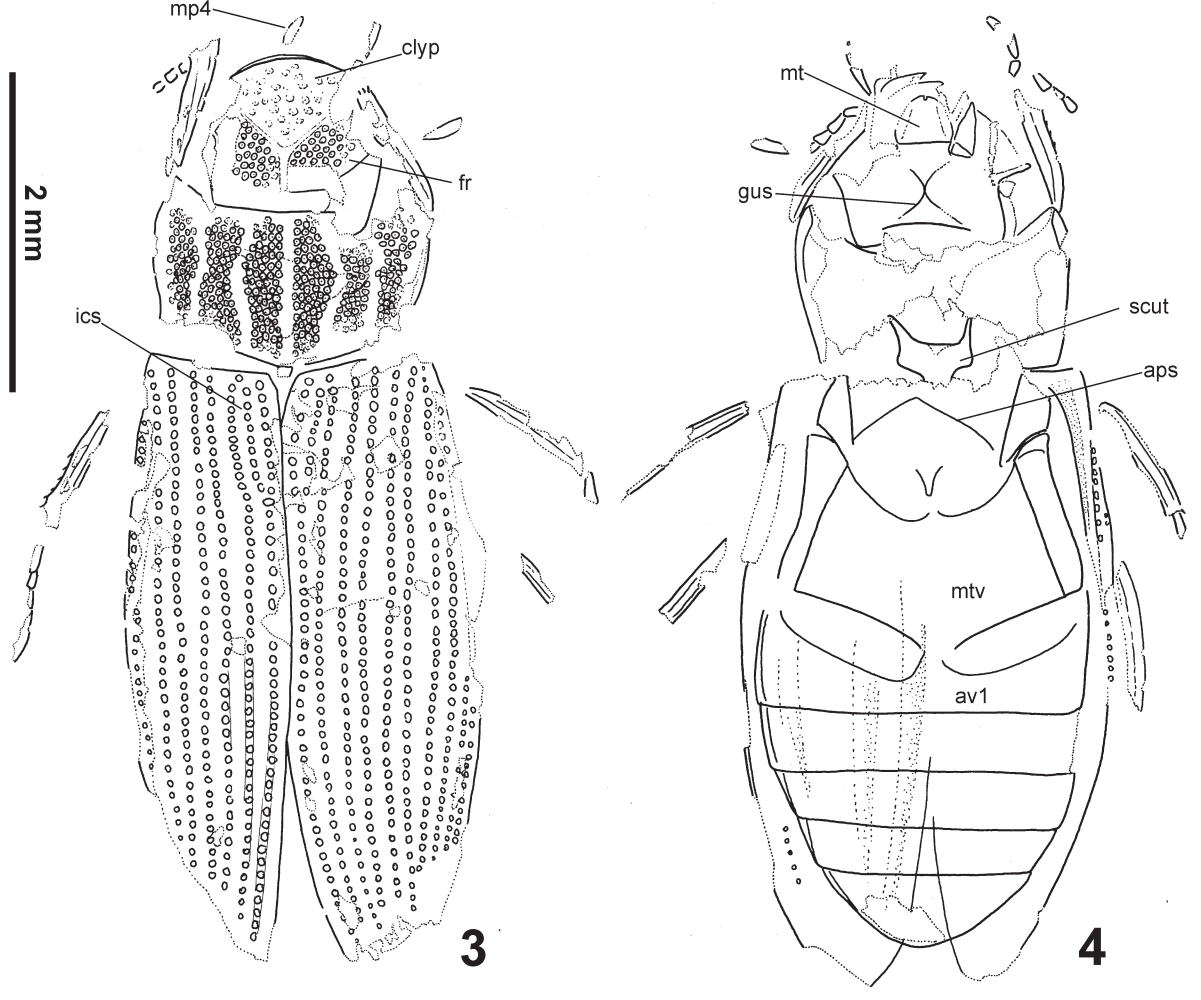
*Helophorus sibiricus* (Motschulsky, 1860), drawings of the early Miocene fossil No. PIN 3285/5 from Kartashevo **3** piece **4** counterpiece. Abbreviations: **mp4** maxillary palpomere 4, **clyp** clypeus, **fr** frons, **ics** intercalary stria, **mt** mentum, **scut** mesoscutellum, **aps** anapleural sutures of mesothorax, **mtv** metaventrite, **av1** abdominal ventrite 1.

(Figs 1–4, 6, 9)**.** Body length 5.76 mm. Head black, with a deeply impressed Y-shaped frontoclypeal suture, basal portion of the groove wide, slightly widened anteriorly. Clypeus with weak remnants of granules only, frons bearing very distinct large setiferous granules isolated by ca. a half of their diameters. Gula strongly constricted behing tentorial pits, gular sutures meeting at one point. Mentum 1.3× wider than long, bearing a deep median emargination on anterior margin (Fig. 6, see the arrow). Apical segment of maxillary palpi asymmetrical. Pronotum1.85× as wide as long, widest at anterior third bearing five wide longitudinal furrows. Bottom of the grooves without granulation. All intervals bearing a uniform, rather dense granulation, granules rather weakly delimited, nearly contacting each other; granulation becoming sparser sublaterally, consisting of several isolated granules along pronotal margins (Fig. 9). Lateral margin regularly convex, not excised subbasally, lacking any apparent tooth-like projections. Pronotal flanks moderately wide anteriorly, slightly narrowing posteriad. Elytrawith 8 preserved series of large rounded to subquadratic punctures (lateral series not preserved due to deformation of elytra during fossilization). Intervals bearing fine and small irregular series of punctures. Scutellar stria present, very long, consisting of 9 punctures on both elytra. Alternate elytral intervals elevate into low ridges (preserved as elongate ridges and furrows on the ventral imprint of the counter-piece). Epipleuron with rather narrow inner pubescent portion, ca. as wide or slightly narrower than elytral flanks. Mesoventritesubtriangular, anapleural sutures nearly straight. Metaventritewider than long, metanepisternum ca. 5.2× as long as wide, with a transverse ridge anteriorly. Abdomenwith five ventrites, ventrite 5 without median emargination, finelly denticulate on whole posterior margin. Legs rather long and slender, protarsi with five tarsomeres.

**Figures 5–9.**
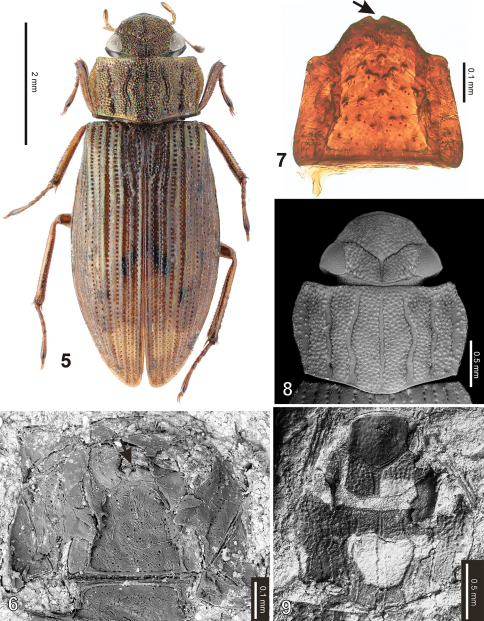
*Helophorus sibiricus* (Motschulsky, 1860) **5** habitus of a recent specimen (Mongolia, Baga-Tenger, coll. National Museum, Prague) **6–7** detail of mentum, the arrow indicates a characteristic emargination on the anterior margin of mentum (**6** fossil specimen, SEM micrograph **7** recent specimen, view from inside) **8–9** detailgranulation of head and pronotum, SEM micrographs (**8** recent specimen **9** fossil specimen).

##### Species attribution.

The subgenus *Gephelophorus* Sharp, 1915 to which we assign the fossil is easily recognizable from other *Helophorus* subgenera by the combination of large body size (4.6–7.0 mm), asymmetrical apical segment of maxillary palpi, elevated alternate elytral intervals, pronotal flanks moderately wide anteriorly and narrowing posteriorly, elytral flanks slightly wider than epipleura. *Helophorus sibiricus* to which we assign the fossil may be recognized from the only other species of *Gephelophorus*, *Helophorus (Gephelophorus) auriculatus* Sharp, 1884, by the continuously curved sides of pronotum not excised behind the anterolateral corners (deeply excised anteriorly and projecting into lateral lobes in *Helophorus auriculatus*), alternate elytral intervals evenly elevated throughout (bearing only isolated elevate tubercles in *Helophorus auriculatus*), and completely and densely granulate pronotal intervals (internal and median interval nearly lacking granules in *Helophorus auriculatus*). Besides, the fossil corresponds with the recent *Helophorus sibiricus* also in other preserved characters: (1) scutellar stria present and very long [absent in several subgenera, extremelly long especially in *Helophorus sibiricus*]; (2) mentum 1.3× wider than long [more than 1.5× as wide as long in *Rhopalohelophorus* Kuwert, 1886, *Helophorus* s.str., and *Lihelophorus* Zaitzev, 1908]. The shape of gular sutures is sexually dimorphic in *Helophorus sibiricus*: the gular sutures are separated throughout in males but meeting in one point in females. Based on this character, we can conclude that the fossil specimen is a female.

##### Recent and fossil distribution

**Figure 10.**
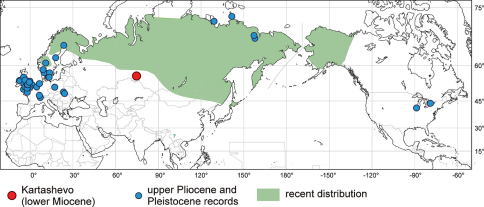
Fossil and recent distribution of *Helophorus sibiricus* (Motschulsky, 1860). Subfossil Holocene records omitted.

(Fig. 10).*Helophorus sibiricus* is at present widely distributed throughout the Holarctic region, from the northern parts of Scandinavia and European Russia through the whole of Siberia and the Russian Far East to Alaska (Angus 1973, 1992, Hansen 2004, Smetana 1985). Its distribution generally corresponds with the range of taiga biome in Eurasia, but slightly exceeds to the temperate and montane forests and grasslands in northern China, Mongolia and Honshu Island, and to the tundra on the north. The species is also reported from Chinese province of Yunnan on the basis of a single historical specimen without precise locality (Angus 1995) – this record may represent a relict mountain population or could be based on mislabeled specimen, and needs to be confirmed by additional material. The northern limit of the distribution of *Helophorus sibiricus* on the Taymyr peninsula is unknown, the northernmost record known to us comes from the environs of Norilsk (S. K. Ryndevich, pers. comm. 2011). In North America, *Helophorus sibiricus* only occurs west of the delta of Mackenzie river and does not reach further east even though both taiga and tundra biomes are present throughout the higher latitudes in Canada. In fossil record, *Helophorus sibiricus* is frequently found in the glacial deposits dated back to late (Devensian/Weichselian) or middle Pleistocene (Saalian) in northern and central Europe (Buckland and Buckland 2006). In North America two known fossil records comes from the late Pleistocene glacial (Wisconsinian) deposits in the Great Lakes area (Morgan and Morgan 1980: Canada, Scarborough; Garry et al. 1990: USA, Illinois). Four records are known from northern Siberian deposits dated back to last glacial (Weichselian; Mamntovy Khayata, Sher et al. 2005), Eemian interglacial (an island in Laptev Sea, Andreev et al. 2004) and late Pliocene to early Pleistocene (Olyorian suite of Krestovka and Chukochya river, Elias et al. 2006). The lower two findings were the oldest records of *Helophorus sibiricus* known so far. The lower Miocene record presented in this paper is situated slightly south of the recent limit of the range of *Helophorus sibiricus*.

##### Biology.

Aquatic species; it is recorded from various kinds of water bodies predominantly with sandy bottom in southern Yamal Penninsula (northeastern Siberia) (Prokin et al. 2008); Angus (1973) considers it as characteristic for river edges in Scandinavia and mentions that it is frequently found in grassy temporary pools particularly those resulting from melting snow in Siberia. Adults of all *Helophorus* speciesare detritivorous. Larvae of *Helophorus sibiricus* are unknown but they may be expected to be terrestrial and predaceous as in most other species of the genus *Helophorus* (Angus 1997).

## Discussion

The genus *Helophorus* is currently divided into 11 subgenera whose monophyly and phylogenetic relationships have not been tested so far. Regardless, its subgroups differ sharply by their morphological and taxonomic diversity. Most *Helophorus* subgenera consist of few species only, each rather distinct morphologically from the others, and are traditionally considered as the remnants of ancient divergences (Fikáček and Angus 2006). On the other hand, a few subgroups (especially the subgenus *Rhopalohelophorus* and parts of the subgenera *Helophorus* s.str. and *Empleurus* Hope, 1838) contain numerous morphologically very uniform species which are sometimes reliably recognizable by few details of morphology and/or karyotypes only (e.g., Angus 1982, 1984, Angus and Aouad 2009, Angus and Toledo 2010). These groups are intuitively considered as results of rather recent diversifications (Fikáček and Angus 2006). *Helophorus sibiricus* represents the first type, as the subgenus *Gephelophorus* only contains two easily distinguishable species at present.

The presented early Miocene fossil of *Helophorus sibiricus* is still too young to be taken as a proof of the relic character expected for *Gephelophorus* due to its high morphological difference but low species diversity. In spite of that, it is the first fossil definitely indicating that at least some small subgenera of *Helophorus* contain species of rather ancient origin rather than recently diverged ones. This seems to contradict the evolutionary scenario proposed for another small and morphologically distinct subgroup within *Helophorus*, subgenus *Kyphohelophorus* Kuwert, 1886, by Matthews (1976). He studied two extinct species of the subgenus (*Helophorus coopei* Matthews, 1976 from late Miocene Lava Camp, and *Helophorus meighenensis* Matthews, 1976 from Meighen Island, both in Alaska) and considered them to be ancestors of the only recent species of the subgenus, *Helophorus tuberculatus* Gyllenhal, 1808. This would indicate either a quick anagenetic change or high divergence and extinction rates of the subgenus during the late Miocene. Recently, Kiselev and Nazarov (2009) recorded *Helophorus tuberculatus* from the late Miocene deposits of Ary-Mas (Taymyr Peninsula, western Siberia) and Letyatkin Cape (north-eastern Siberia). If the identification of these fossils is correct, they would show that all three *Kyphohelophorus* species lived at the same time, proposing a scenario different from Matthews’ (1976) one: all three *Kyphohelophorus* species may originate during the Miocene (or earlier) but only *Helophorus tuberculatus* survived until present. *Helophorus sibiricus* might fit a similar scenario based on the fossil presented within this paper.

The Miocene record of *Helophorus sibiricus* presented in this paper is not the only pre-Pleistocene record of recent hydrophiloid beetles. Hayashi (2001) and Hayashi et al. (2003) recorded modern Japanese species *Coelostoma stultum* Walker, 1858, *Coelostoma orbiculare* (Fabricius, 1775), *Hydrochara libera* (Sharp, 1884), *Sternolophus rufipes* (Fabricius, 1792) and *Regimbartia attenuata* (Fabricius, 1801) from the Japanese early Pliocene (Tsubusagawa Formation) and late Pliocene deposits (Uonuma, Ookui and Oizumi Formations). Unfortunately, all these taxa were only found as isolated elytra and pronota lacking any species-specific diagnostic characters, and it is therefore impossible to imply if the fossils really represent recent taxa. Similar uncertainty concerns the late Miocene records listed by Kiselev and Nazarov (2009), identified as *Helophorus tuberculatus* (mentioned above) and *Coelostoma orbiculare*, as no details on the morphology of the fossils are provided. On the other hand, the morphology of the early Miocene fossil of *Hydrophilus* cf. *pistaceus* Castelnau, 1840 was studied in detail by Fikáček et al. (2008), but the preserved morphological characters only allowed for its approximate identification. Although all these records indicate that the Miocene age may not be exceptional for a hydrophiloid species, the early Miocene fossil of *Helophorus sibiricus* presented here represent the first hydrophiloid fossil of this age in which the preserved characters allow a reliable identification. Early Miocene (i.e., ca. 16–23 mya) may be thus considered as the maximum age of a recent hydrophiloid species known at present. In contrast, all well-preserved late Oligocene and older fossils studied by us so far were found to belong to extinct species (Wedmann 2000, Fikáček et al. 2010a, b, c).

The habitat as well as climatic requirements of the beetle species are usually considered stable over the time (Coope 2004, Elias 2007, Hörnschemeyer et al. 2010), which provides three possible ways to explain the survival of these species since the Tertiary: (1) life-style of the species is associated with a habitat which is stable over the time, (2) the species was able to track suitable environmental conditions though the climate changed over the time; (3) the species is surviving in a single area with stable environmental conditions over several millions of years. Although the generalized aquatic life-style makes the habitat-based explanation seemingly improbable for *Helophorus sibiricus*, the occurrence of the notostracan “living fossils” in temporary pools (Mantovani et al. 2004) indicates that this kind of waters may provide stable conditions over a geological time for taxa adapted for their seasonality. *Helophorus sibiricus* is also able to track suitable environmental conditions when climate is changing: its distribution was largely affected by the Pleistocene climate changes even though it presently inhabits an area with wide range of climatic conditions (mean January temperature below –2°C, mean July temperature varying between 3–14°C; S. Elias, pers. comm.). The survival of *Helophorus sibiricus* in the long-lasting stable environment in south-western Siberia cannot be excluded either as the environment in many parts of Central Asia was shown to remain extremely stable at least since the Pleistocene (e.g., Chytrý et al. 2010). The early Miocene is characterized by the formation of the first stable grasslands alternating with coniferous-small leaved forests in south-western Siberia (Velichko and Spasskaya 2002), which may correspond with the recent environment inhabited by *Helophorus sibiricus* in the southern part of its range. Hence, we cannot exclude that some locally limited ecosystems of south-western Siberia might have been stable enough since the late Tertiary, allowing the survival of *Helophorus sibiricus* and other Tertiary species.

## Supplementary Material

XML Treatment for Helophorus (Gephelophorus) sibiricus
